# Statin-Intolerant Patients Exhibit Diminished Muscle Strength Regardless of Lipid-Lowering Therapy

**DOI:** 10.3390/jcm14041221

**Published:** 2025-02-13

**Authors:** Pierandrea Vinci, Filippo Giorgio Di Girolamo, Federica Pellicori, Emiliano Panizon, Alessia Pirulli, Letizia Maria Tosoni, Nicola Altamura, Stefania Rizzo, Andrea Perin, Nicola Fiotti, Gianni Biolo

**Affiliations:** 1 Unità Clinica Operativa Clinica Medica, Department of Medical, Surgical and Health Sciences, Faculty of Medicine, University of Trieste and ASUGI, 34149 Trieste, Italy; federica.pellicori@asugi.sanita.fvg.it (F.P.); emiliano.panizon@asugi.sanita.fvg.it (E.P.); alessia.pirulli@asugi.sanita.fvg.it (A.P.); letizia.tosoni@gmail.com (L.M.T.); nicola.altamura@asugi.sanita.fvg.it (N.A.); sterizzo.93@gmail.com (S.R.); a.perin85@gmail.com (A.P.); fiotti@units.it (N.F.); biolo@units.it (G.B.); 2 Hospital Pharmacy, Cattinara Hospital, Azienda Sanitaria Universitaria Giuliano Isontina, 34149 Trieste, Italy; fgdigirolamo@gmail.com

**Keywords:** statin intolerance, BIA, skeletal muscle mass, muscle quality, handgrip, arm dominance, hypercholesterolemia, creatinine kinase

## Abstract

**Background and Aims:** Statin-associated muscle symptoms (SAMS) is a frequent side effect of statin therapy, limiting its clinical use and increasing cardiovascular risk. Its relationship with muscle performance and quality is not completely understood. The aim of our study was to retrospectively assess the differences between body composition and muscle strength in patients with SAMS, compared with matched controls. **Material and Methods:** cardiovascular risk factors, lipid profile, and body mass index (BMI), were analyzed in 148 statin-intolerant (SI) and in 145 sex- and age-matched statin-tolerant (ST) patients attending a secondary-level outpatient lipid clinic. At the end of follow-up (mean 45 months), the evaluations were reassessed and bioelectrical impedance analysis (BIA)-assessed body composition, and muscle quality (handgrip/skeletal muscle mass) were further determined. **Results:** At baseline, BMI, cholesterol, and triglycerides in SI were higher than in ST patients. During follow-up, SI patients underwent a further increase in BMI and low-density lipoproteins (LDL)-cholesterol remained significantly higher than in ST patients. At the end of the follow-up, BIA-assessed fat mass percentage was higher in SI than in ST. Handgrip absolute values or standardized for skeletal muscle mass (muscle quality) were significantly lower in SI patients (*p* < 0.001), but this was confirmed only in their non-dominant arm (*p* < 0.01 for all arms). Circulating creatine kinase levels, which was higher in SI patients at baseline (*p* < 0.001), remained higher in those who never restarted statins after re-challenge (*p* = 0.029). **Conclusions:** Statin intolerance is clinically associated with lower muscle quality, particularly in less exercised arms.

## 1. Introduction

Statins (3-hydroxy-3-methylglutaryl-Coenzyme A reductase inhibitors) are the first line of treatment for dyslipidemia in primary and secondary prevention of cardiovascular diseases. The main limitation of this strategy is statin-associated muscle symptoms (SAMS), a clinically heterogeneous condition which encompasses symmetrical muscle pain, weakness, and fatigue; frequently appearing early after the beginning of statin treatment [1], with occasional rhabdomyolysis, and with or without changes in circulating creatine kinase (CK) [2]. Diagnosis of SAMS may be difficult and it is actually based on the SAMS-Clinical Index (SAMS-CI) classifying the origin of muscle pain according to whether it is “unlikely”, “possibly”, or “probably” related to statin therapy [3,4]. Prevalence of the condition seems to be around 2–3% of the general population and, according to a recent meta-analysis of 176 studies and 4,143,517 patients with dyslipidemia, 9.1% worldwide [5]. Skeletal muscle being a target of statin intolerance, this drug might account for muscle decline and triggering of sarcopenia. Anthropometric measures, such as body mass index (BMI) and waist circumference (WC), are associated with body fat quantity and distribution. However, they do not inform about patients’ body composition, particularly their skeletal muscle mass (SMM) quantity and quality. Bioelectrical impedance analysis (BIA) is an indirect method which performs a quantitative and qualitative analysis of body composition; this instrument, indeed, through the induction of a low-frequency current beam, allows the determination of body mass and composition and a precise estimate of the body’s hydration status [6]. Functional muscle quality, expressed as muscle quality index (MQI)—i.e., the ratio between handgrip (HG) and SMM—represents a current methodology to measure the capacity to generate force [7]. The possible role of sarcopenia as a risk factor for SAMS is debatable, since the literature on this subject provides conflicting results, with some studies showing strength reduction [8,9,10], and others either strength increase [11] or no differences at all [12,13] in statin use. The presence of weakness plus asymmetry may represent a more advanced stage of muscle function impairment than either condition alone [14]. In itself, asymmetry, as another potential indicator of impaired muscle function, has been associated with the increased risk of developing comorbidities during aging, and worsening the functional disability, which means losing the ability to perform activities of daily living (ADLs) [15]. A less than 10% strength difference between the dominant and non-dominant arms can be considered physiological [16]. The dominant hand usually exerts more strength and provides motor performance with greater skill than the non-dominant hand. Therefore, the non-dominant arm and hemisoma, may have increased the risk of developing musculoskeletal and neuromuscular disorders [17]. Limitations of the currently available studies on muscle efficiency/weakness during statin therapy are their small size, lack of a precise criteria and a validated test or biomarkers for its diagnosis; with controversial results on the few papers written on muscle quality and performance in statin intolerance.

The primary aim of our study was to compare body mass index and lipid profile changes over time in two age- and sex-matched groups of dyslipidemic patients being either statin tolerant (ST) or intolerant (SI). The secondary aim was to compare muscle quality (expressed as ratio between muscle strength and mass) and body composition determined at the end of the follow-up between SI and ST patients.

## 2. Materials and Methods

### 2.1. General Plan of the Study

**Figure 1:**
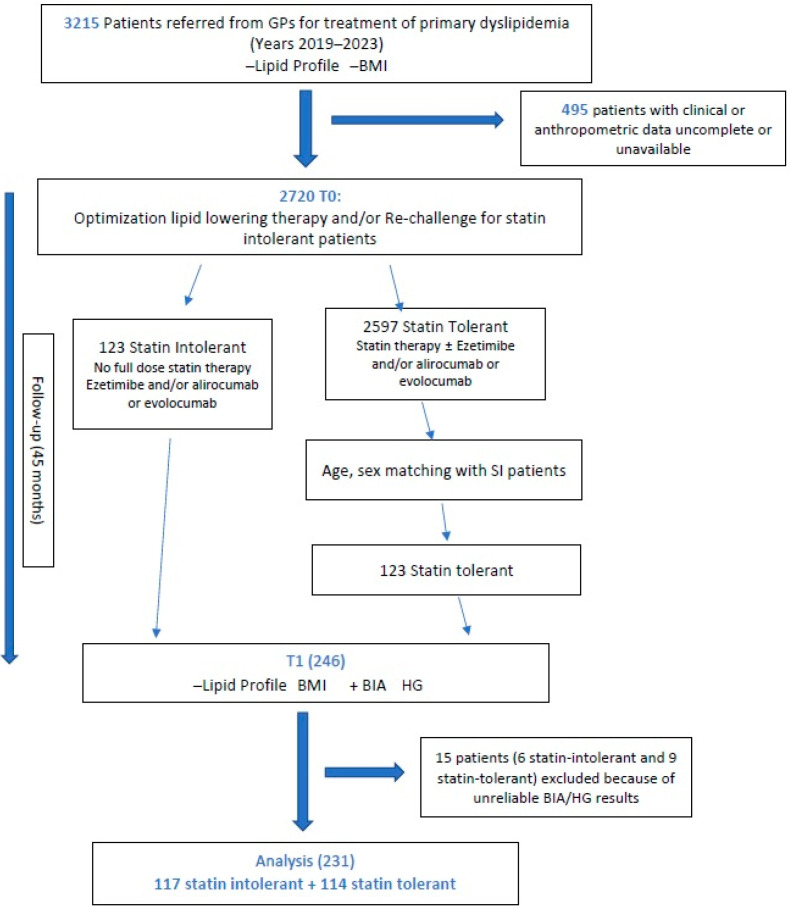
Flowchart of the study. Legend = BMI: body mass index, BIA: bioelectrical impedance analysis, HG: handgrip.

All cases included in this study were selected from the medical records of patients attending the secondary-level outpatient clinic for dyslipidemias (Clinica Medica Unit, ASUGI/University Teaching Hospital of Trieste, Trieste, Italy) on the basis of tolerance to statins. The general plan of the study is shown in Figure 1.

The observation period started with the first visit in which statin intolerance was diagnosed, or with the first attending clinic visit in which lipid profile and body mass index (BMI) were assessed. Patients who did not have their BMI measured, or did not have complete clinical or anthropometric data were excluded from the study. At enrolment (T0), optimization of lipid-lowering therapy and/or re-challenge for SI patients were performed. At that time, the enrolled patients were divided in statin intolerant and statin tolerant groups. Randomization was carried out, dividing ST patients according to sex and age (listed in alphabetical order) and matching each SI patient with the first patient of the same sex and age (±2 year) from the ST list.

The observation period ended at T1 (median value 45 months after enrolment), when lipid profile and body mass index (BMI) were reassessed, and a bioelectrical impedance analysis (BIA, with BMI assessment), and handgrip (HG) evaluation were performed. At this time, patients were matched for sex and age; therefore, 231 patients were ultimately evaluated. Exclusion criteria were age < 18 years old, patients with secondary hyperlipidemias, and a refusal to a sign written informed consent. Patients who did not have a BMI, BIA, and HG performed were excluded.

### 2.2. Baseline Observation (T0)

At arrival to the center, the patients underwent an optimization lipid profile, a routine laboratory test including muscle and liver enzymes (CK and aminotransferases), anthropometric evaluation (BMI), and collection of personal and family history (encompassing Dutch Lipid Clinic Network criteria). Moreover, screening for statin intolerance was carried out with the SAMS-CI and data about the kind of statin, dosage, treatment regimen, and the cytochrome involved in the statin metabolism were collected. We considered intolerant those patients who were classified as “possibly” or “probably” affected, according to SAMS-CI. The patients were then regularly and periodically recalled for follow-up examinations until April 2023. The patients, who may or may not have been on statin therapy upon arrival at the center, were standardized to T0.

### 2.3. Follow-Up Examination (T1)

In April 2023, the parameters obtained at T0 were repeated and completed with BIA and a handgrip test. The BIA Nutrilab (Akern, Italy) was used according to the standard protocol; patients had an empty stomach and bladder during the examination. The handgrip analysis was obtained using a Lafayette Hydraulic Hand Dynamometer; both hands were tested twice, keeping the highest result for each hand. In evaluation of muscle quality and performance, the ratio between muscle strength (HG expressed in kg) and the skeletal muscle mass (SMM) extrapolated from the BIA (HG/SMM) and the ratio between muscle strength and skeletal appendicular muscle mass (ASMM) (HG/ASMM) were obtained.

The study complies with the Helsinki criteria and was approved by the Ethical Committee of the Friuli Venezia Giulia Region CEUR (n° 28/2017) on 13 July 2017.

## 3. Statistical Analysis

The data are described as median and interquartile range for continuous variables or as absolute figures for categorical ones. Differences in continuous variables between intolerant patients or controls were assessed by nonparametric statistics (using the Mann–Whitney U or Kruskal–Wallis tests when appropriate), while categorical variables were compared using the chi-square test and, when suitable, with hazard ratio and 95% confidence interval. For variables assessed more than once (lipid profile, BMI, enzymes), the generalized linear model (GLM) method for repeated measures was adopted; considering statin tolerance, current drug use status or, for HG, the dominant arm vs. the non-dominant arm as distinguishing factors between subjects. The null hypothesis was rejected when the *p* value was less than 0.05. Statistical analysis was performed using SPSS software version 21.0.

## 4. Results

Out of 3215 patients referred from a general practitioner for treatment of primary dyslipidemia to our outpatient clinic, the number of patients with a usable/complete dataset was 246, after sex and age standardization. We excluded 15 patients (6 statin-intolerant and 9 statin-tolerant) because of unreliable BIA/HG results; hence, the final figures were 117 intolerant and 114 tolerant patients. The study design is reported on the flowchart (Figure 1) and all statistics and comparisons refer to these two groups.

### 4.1. Baseline Data Analysis (T0)

At T0 analysis, triglycerides, CK, and creatinine (*p* = 0.011, 0.003 and 0.042, respectively) were higher in SI patients. It should be noted that there was a trend towards a higher BMI in SI patients. A sub-analysis considering three groups of statin intake—i.e., tolerant, intolerant who assumed statins, and intolerant patients who did not reassume statins after re-challenge—was conducted, but the results overlapped with the comparison between the two groups above. Detailed results are reported in Table 1 and in Supplementary Table S1.

### 4.2. Follow-Up Analysis (T1)

At T1, SI patients showed a higher body mass index (BMI) and higher levels of total cholesterol, LDL cholesterol, and triglycerides (*p* = 0.005, 0.001, 0.001, and <0.001, respectively) than ST patients. Concerning BMI changes, the difference between tolerant and intolerant patients was significant when comparing the absolute value, the percentage change compared to baseline, and also when standardized by follow-up duration (Table 2).

At the BIA analysis, SI patients had a greater fat mass amount, and a lower percentage of fat-free mass and total body water loss (*p* = 0.010, 0.032, and 0.025, respectively) (Supplementary Table S2). Even if we found the same trend in female subjects, the male subjects drove the statistically significant difference.

### 4.3. Strength and Muscle Quality

**Figure 2:**
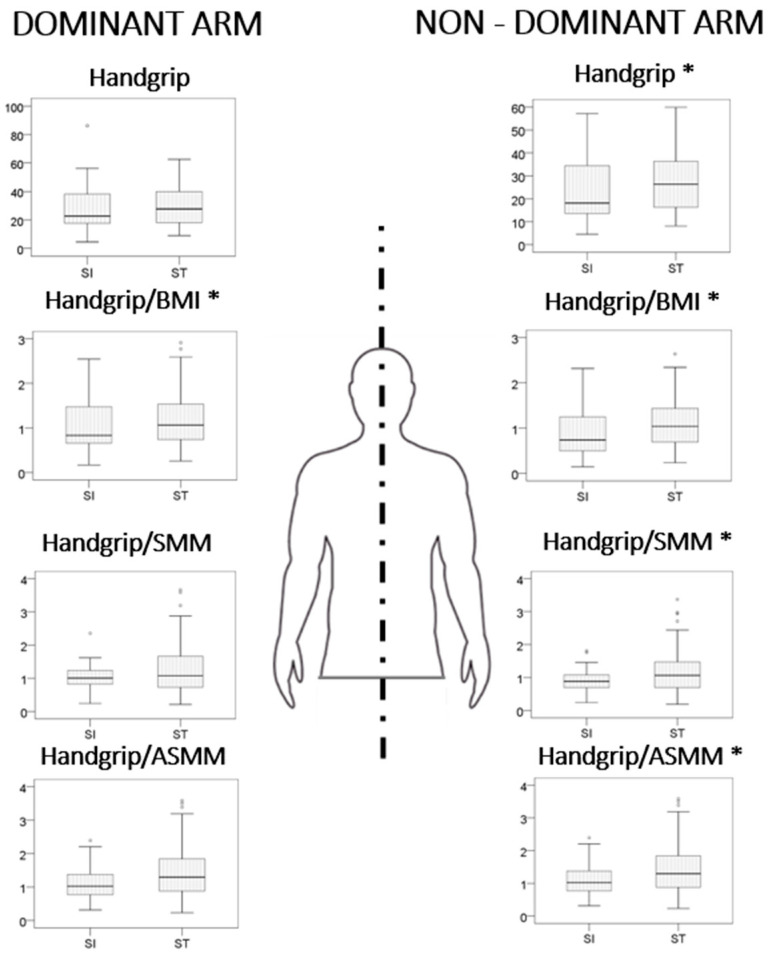
Differences in HG and MQi between dominant and non-dominant arms. Mann–Whitney U test * *p* < 0.05.

In a sub-analysis conducted according to the dominant or the non-dominant arm, all these markers were significantly lower only in the non-dominant arm. The HG ratio between the dominant and non-dominant arm is significantly different between the two groups (*p* = 0.043); in the SI group the difference between dominant and non-dominant HG was 11.3%, while in the ST group it was 7.5%. Considering a physiological <10% difference in HG between the two arms, the prevalence of patients with >10% was significantly higher in the SI group than in the ST group (56.6 vs. 41.1, respectively, OR 1.87 95% CI 1.08–3.23, *p* = 0.025) (Figure 2).

Differences in absolute handgrip values and muscle quality (standardized by BMI, SMM, and ASMM) between SI and ST patients remained significant also when patients with previous vascular events (index of lower muscular fitness) were excluded. Values of absolute HG and muscle quality in the dominant and non-dominant arms are detailed according to sex in Supplementary Table S3.

**Figure 3:**
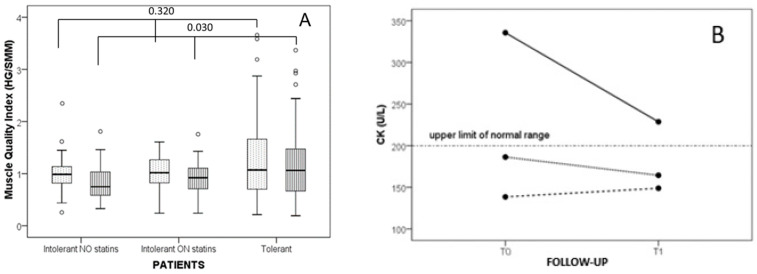
Muscle quality index and time course of CK in patients according to tolerance and use of statins. (**A**): Muscle quality index (HG/SMM) is divided according to the dominant (lightly dotted) or the non-dominant (vertical lines) arm (Kruskal–Wallis test *p* = 0.32 and 0.03 for dominant and non-dominant, respectively); (**B**) time course of the CK according to the statin tolerance. Differences in CK between the first observation and the actual therapy according to the tolerance of patients, Generalized Linear Model *p* = 0.029. Legend: solid line: Intolerant NO statin; dotted line: Intolerant ON statins; dashed line: Tolerant.

Lipid-lowering treatment among SI patients is associated with the muscle quality, with a lower HG/SMM ratio in patients avoiding statins (Figure 3A). Such a difference was not statistically significant in a post hoc analysis test and the direct comparison of SI patients with NO statins vs. all the other patients grouped yielded a trend towards lower quality (*p* = 0.067). CK levels, measured at T0 (during usual therapy or re-challenge) and reassessed at T1, shows—in SI patients not assuming any statin—an important reduction during follow-up, but not reaching normal levels; while the other two groups were roughly unchanged (GLM *p* = 0.004, interaction of time course between patients intolerant not on statins vs. all patients on statins *p* = 0.029, Figure 3B).

## 5. Discussion

In this study, statin-intolerant patients attending a secondary-level outpatient clinic for dyslipidemia treatment had higher BMI at arrival, which further increased at follow-up compared to sex- and age-matched statin-tolerant controls with polygenic hypercholesterolemia. Creatinine kinase levels were significantly reduced in intolerant patients during follow-up after changes in therapy, although the final levels at the end of follow-up remain higher than normal. Finally, in a cross-sectional evaluation, muscle quality was lower in intolerant patients, independent of the possibility to assume reduced amounts of statins.

Intolerant patients show higher BMI levels at baseline. Furthermore, statin intolerance is associated with significant weight increase over the years of observation, and BIA at the end of follow-up found a higher fat mass (FM) and fat mass index (FMI), even when standardized by the duration of follow-up. In intolerant patients, BMI increase was not statistically different between statin users and statin avoiders, in spite of an important difference between the two groups; this is probably due to the low numerosity of statin avoiders. Looking at the median value, it can be argued that statin-intolerant patients who cannot stand even the lowest statin dose increases his/her body mass by around half a BMI unit per year. This finding seems in line with a recent review that found augmented adiposity, especially visceral, in patients with SAMS [18].

The most likely explanation for this increase in BMI in SI patients is that statin-induced muscular pain triggers avoidance behavior—i.e., reduced physical activity—which, in turn, accounts for this increase in fat accumulation. This effect would therefore be independent of statin use, and lack of a reliable assessment of physical activity between the two groups prevents us from drawing a firm conclusion. Alternatively, other explanations are that obesity might be in itself a risk condition triggering inflammation [18], or that statins interfere with the endocannabinoid pathway, inducing fat accumulation [19]. The widespread belief that statins cause muscle symptoms enhances the nocebo effects in many statin users [20] and the contribution of these patients to the study cannot be ruled out. As a matter of fact, a gold standard definitive diagnostic criteria or test for SAMS is lacking; creatinine kinase levels are frequently normal in symptomatic patients assuming statins, while they may be increased in asymptomatic patients [21]. Phillips et al. [9] reports that statin-induced myopathy often occurs in the presence of normal serum creatinine kinase activity, concluding that its levels are not reliable indicators of myotoxicity [22]. Clinical trials commonly define statin-induced toxicity as myalgia or muscle weakness with creatinine kinase levels greater than 10 times the normal limit [23,24,25]. In a meta-analysis of 16 studies including 41,457 patients by Kashani et al. [26] the elevation of this enzyme was not significantly higher in patients treated with statins [27]. On the relationship between fat accumulation and creatinine kinase levels, our study allows us to put forward two hypotheses. The first is that patients not tolerating any statin dosage are intrinsically prone to both BMI increase and to a kind of myopathy that becomes symptomatic after statin use; or, second, that in these patients (with normal BMI and CK), statin use triggers a self-maintaining condition that continues even after statin suspension.

It is worth mentioning the slightly higher creatinine levels in intolerant patients, which does not have an obvious explanation. The matching of age and gender rules out these two variables as possible causes of this difference. The correlation between CK and creatinine at T1 but not at T0 (data not reported) leaves room to speculate that this small difference could not only reflect kidney function, but it could also indicate muscle cell damage. This hypothesis needs to be verified with a more focused study.

More obvious is the increase in triglycerides, which could reflect the lower physical activity in intolerant patients.

The last finding is a reduced physical fitness in patients with statin intolerance when evaluated with a performance-based test, and this effect was mainly observed in the non-dominant and less exerted forearm. Muscle functional status assessment—i.e., mass and performance—can be evaluated in several ways. Muscle mass can be quantified by computed tomography (CT) or magnetic resonance imaging (MRI), which are currently considered the gold standards; or dual-energy X-ray absorptiometry (DEXA), or BIA [28]. Handgrip has been widely used as a good indicator of total body strength, as well as being easy to use and access [29,30,31], although the lower limbs are more relevant than the upper ones in the physical functions of everyday life. Handgrip is the force parameter we used for calculating muscle quality index (MQi), since it is a simple method that is easily applicable in clinical practice and correlates with total muscle strength [32]. Independent of intolerance, statin is associated with a slight worsening of the muscle quantitative and qualitative parameters and an increase in muscular adipose tissue (MAT) in a 4-year follow-up observational MRI-based study [33]. It is possible that the accumulation of MAT with the resulting percentage reduction in the contractile component causes the muscle strength deficit associated with statin use. Concerning muscle function, there are few studies that have actually evaluated this condition in a quantifiable way and the results were not consistent [34,35,36,37]. In our study, handgrip performance in the recessive arm of intolerant patients was significantly lower than that observed in tolerant patients, while the dominant arms seem unaffected. To rule out possible bias or confounders in analysis, the handgrip results have been standardized by muscle mass in order to detect the muscle quality, defined as the ratio of muscle strength to the unit of muscle mass, either skeletal appendicular mass, muscle volume or skeletal muscle mass [35,36] and referred as the MQi. Indeed, a standardized method for calculating MQi has not yet been validated, while different approaches have been proposed, so far: one repetition maximum (1RM) divided by the muscle mass of the limb used, the combination of extensor and knee flexor divided by muscle mass, the strength of the handgrip divided by muscle mass (MM), skeletal muscle mass (SMM), or appendicular muscle mass (ASMM) [38]. In our study, there were significant differences in terms of MQi, expressed in two different methods/ratios and strength/mass. In the dominant arm the trend was similar, but not statistically significant. It could be hypothesized that low MQi is a risk index for statin intolerance. The most likely explanation is that the amount of exercise loads in dominant muscle improved muscle quality. Some studies, accordingly, suggest that the evaluation of strength asymmetry could implement the evaluation of functional performance, independent of the trigger, and therefore improve the definitions of sarcopenia and dinapenia [14]. To date, studies regarding asymmetry focus mainly on age-related decline in muscle function and, to our knowledge, there are no data in the literature on statin intolerance.

## 6. Limitations

In our study, patients considered to be intolerant were all those who—due to reported muscle symptoms—had to discontinue statin therapy, either permanently undergoing re-challenge or de-escalation. Not all of these patients had a high SAMS-CI score, and this may be a limitation of the study. National Lipid Association guidelines on statin intolerance encompass both patients with a high SAMS score and those requiring de-escalation. A limitation of our study is that we have bioimpedance and HG data at T1 but not at enrolment, so it was not possible for us to compare body composition and strength at the two different time points. We, therefore, only consider BMI changes and it remains unknown how statin therapy modifies muscle strength. Furthermore, we did not administer any questionnaires, so we have no data on physical activity and diet, which would have helped us to know the origin of fat mass increase in these patients.

## 7. Conclusions

In a longitudinal, 3.5-year observational study, fat mass in statin-intolerant patients with dyslipidemia increased faster than it did in tolerant ones. In addition, muscle quality (strength/mass) in the arms follows a pattern resembling the probable utilization; which is highest in the dominant arms of tolerant male patients and lowest in the non-dominant arms of intolerant female patients. In intolerant patients who did not tolerate any statin regimen, high circulating creatinine kinase activity did not normalize after 3 years following the suspension of the drug.

## Figures and Tables

**Table 1:** Population at baseline. Legend: SI: statin intolerant; ST statin tolerant; BMI: body mass index; N: non-smoker; EX: former smoker; CUR: current smoker; Lp(a): lipoprotein(a); HbA1c: glycated hemoglobin; HDL: high-density lipoproteins; LDL: low-density lipoproteins; ALT: alanine transaminase; AST: aspartate aminotransferase; CK: Creatinine Kinase; TSH: thyroid stimulating hormone.

	SI Patients (*n* = 117)	ST Patients (*n* = 114)	*p* (Mann–Whitney U Test)
Anthropometric data			
Male/female	56/61	53/61	
Age (years)	63 (57–70)	62 (56–68)	0.296
Height (cm)	168 (161–176)	167.5 (162–176)	0.321
Weight (kg)	74.5 (65–84.6)	71 (61–84)	0.317
BMI (kg/m^2^)	26.44 (23.5–29.33)	24.92 (23.11–27.94)	0.054
Diabetes (*n*)	12	9	0.532
Hypertension (*n*)	35	29	0.447
Smoking habit (N/EX/CUR)	92/16/9	80/18/16	0.237
Duration of follow-up (days)	1377 (812–19,612)	1220 (403–2435)	0.833
Laboratory results			
Creatinine (mg/dL)	0.91 (0.77–1.03)	0.86 (0.71–0.96)	0.042
PCR (mg/L)	1.1 (0.6–2.5)	0.6 (0.4–1.3)	0.130
Total cholesterol (mg/dL)	245 (192–280)	239 (197–277)	0.706
Triglycerides (mg/dL)	130 (103–183)	108.5 (84–164)	0.011
HDL (mg/dL)	54 (48–67)	57 (48–71)	0.118
LDL (mg/dL)	144.7 (109.5–183)	154.8 (110, 182)	0.843
Non-HDL (mg/dL)	229 (171–254)	212 (157–241)	0.268
Lp(a) (mg/dL)	16.7 (5–59.5)	30.6 (5–87)	0.193
CK (U/L)	163.5 (100–241)	126.5 (95.5–164.5)	0.003

**Table 2:** BMI variations during Follow-up. Legend: SI: statin intolerant; ST statin tolerant; NO statins: SI patients not assuming statins; ON statins: SI patients assuming statins; BMI: body mass index; Delta BMI: BMI*T1*–BMI*T0*; Delta BMI %: Comparison has been carried out with Kruskal–Wallis test.

	SI NO Statins (*n* = 26)	SI ON Statins (*n* = 85)	ST Patients (*n* = 102)	*p*
Delta BMI	0.53 (1.85–−0.03)	0.2 (1.36–−0.44)	0.006 (0.72–−0.7)	0.009
Delta BMI %	0.24 (0.58–−0.02)	0.06 (0.28–−0.12)	0.00 (0.18–−0.24)	0.004
Delta BMI/year	0.24 (0.84–−0.01)	0.01 (0.6–−0.03)	0.005 (0.31–−0.32)	0.011
Delta BMI %/year	0.11(0.24–−0.01)	0.03(0.15–−0.06)	0.0(0.08–−0.12)	0.007

## Data Availability

The data included in this study are available from the corresponding authors upon reasonable request.

## Supplementary Materials

The following supporting information can be downloaded at: https://www.mdpi.com/article/10.3390/jcm14041221/s1. Table S1: Risk factors and comorbidities in the study group. Legend: SI = statin intolerant, ST = statin tolerant, P = Pearson Chi-square. Table S2: BIA results. Legend: SI = statin intolerant, ST statin tolerant, Rz: resistance; Xc: reactance; FFM: fat free mass; TBW: total body water; ECW: extracellular water; BCM: body cell mass; FM: Fat Mass; PA: Phase angle; MM: Muscle Mass, SMI: Skeletal Muscle Index; SMM: Skeletal Muscle Mass; ASMM: appendicular skeletal muscle mass; FMI: Fat mass index; FFMI: Fat free mass index. Table S3: Differences in HG and MQi between dominant and non-dominant arms. Results are expressed as median and IQR values. P = Kruskal-Wallis test. A: results in the whole population. B: results in Men; C: results in Women; Legend: HG: Hand Grip; SMM: Skeletal Muscle Mass; ASMM: appendicular skeletal muscle mass; BMI: body mass index.
