# Supplementary material for: Statin-Intolerant Patients Exhibit Diminished Muscle Strength Regardless of Lipid-Lowering Therapy
Source: J Clin Med. 2025 Feb 13;14(4):1221. doi: 10.3390/jcm14041221 (PMC11856913; doi:10.3390/jcm14041221)
Supplement: Supplementary file 1 [file jcm-14-01221-s001.zip › jcm-3451353-supplementary.pdf]

	SI		ST	
	SI NO statins	SI ON statins	ST patients	P
Family history of diabetes	4	3	16	0,037
Family history of hypertension	0	2	13	0,012
Family history of dyslipidemia	17	46	78	0,094
Family history of cardiovascular disease	4	18	24	0,677
Diabetes	5	7	9	0,263
Hypertension	11	24	29	0,393
Hypothyroidism	4	12	14	0,890
Chronic renal failure	1	3	2	0,716
Atherosclerotic disease	8	14	8	0,001
Liver disease	1	2	7	0,411
Myopathy	0	1	0	0,436
Active smoking (never/former/current)	21/6/2	71/10/6	80/18/17	0,224
Alcoholism	0	0	1	0,600

Supplementary material Table S1. Risk factors and comorbidities in the study group. Legend: SI= statin intolerant, ST =statin tolerant, P=Pearson Chi-square

	SI patients (n=117)	ST patients (n= 114)	P
Rz	491 (440.1-556.7)	491.1 (436.85-559.25)	0.845
Xc	53.9 (50.3-59.1)	53 (47.25-60.9)	0.390
FFM (Kg)	52.7 (46.7-65.2)	52.4 (44.65-66.25)	0.921
TBW (Kg)	38.7 (34.2-47.6)	38.6 (32.8-48.75)	0.966
ECW (Kg)	17.6 (15.9-19.7)	18 (15.15-20.95)	0.796
BCM (Kg)	29.1 (24.3-37.5)	28.9 (23.8-38.3)	0.683
FM (Kg)	18.8 (13.5-24.4)	17.1 (12.65-21.5)	**0.010**
PA (°)	6.2 (5.6-7)	6.2 (5.7-6.5)	0.312
FM (%)	25.6 (19.4-33.5)	23.8 (17.8-27.85)	**0.032**
FFM (%)	74.4 (66.5-80.6)	76.2 (72.15-82.2)	**0.032**
TBW (%)	54.6 (48.7-58.9)	56.1 (52.9-60.3)	**0.025**
ECW (%)	45 (41.7-47.6)	45 (43.5-47.1)	0.315
ICW (%)	55 (52.4-58.3)	55 (52.9-56.5)	0.315
MM (Kg)	23.7 (19.2-32)	24.5 (19.3-33)	0.526
MM (%)	35 (27.8-39.7)	35.1 (31.2-40.45)	0.054
SMI (Kg)	9 (7.4-10.5)	8.9 (7.4-10.55)	0.978
SMM (Kg)	23.7 (19.2-32)	24.5 (19.3-33)	0.526
ASMM (Kg)	19.8 (17.2-24.7)	20.1 (16.4-25.8)	0.939
FMI	6.6 (4.5-9.2)	5.9 (4.2-7.5)	**0.009**
FFMI	19.6 (17.8-21.4)	19.3 (17.4-21.35)	0.279

Supplementary material Table S2. BIA results. Legend: SI= statin intolerant, ST statin tolerant, Rz: resistance; Xc: reactance; FFM: fat free mass; TBW: total body water; ECW: extracellular water; BCM: body cell mass; FM: Fat Mass; PA: Phase angle; MM: Muscle Mass, SMI: Skeletal Muscle Index; SMM: Skeletal Muscle Mass; ASMM: appendicular skeletal muscle mass; FMI: Fat mass index; FFMI: Fat free mass index.

A.

	Intolerants	Tolerants	P
Age	57 (63-69,5)	56 (62-68)	
HG dominant (Kg)	18,14 (24,49- 39,01)	18,14 (28,12 39,92)	**0.183**
HG recessive (Kg)	14,06 (19,96-34,92)	17,24 (27,22-36,74)	**0.048**
HG/SMM dominant	0,83 (1,012-1,25)	0,77 (1,1-1,72)	**0.162**
HG/SMM recessive	0,70 (0,89-1,09)	0,72 (1,06-1,6)	**0.011**
HG/ASMM dominant	0,98 (1,22-1,61)	0,96 (1,34-2,11)	**0.080**
HG/ASMM recessive	0,80 (1,03-1,38)	0,88 (1,32-1,9)	**0.005**
HG/BMI dominant	0,66 (0,89-1,49)	0,76 (1,12-1,53)	**0.027**
HG/BMI recessive	0,51 (0,76- 1,28)	0,7 (1,06-1,47)	**0.004**

B.

	Intolerants	Tolerants	P
Age	53 (60-68)	55 (59,5-68)	
HG dominant (Kg)	31,75 (39,01-45,36)	19,05 (27,67-38,1)	**0.000**
HG recessive (Kg)	27,22 (34,92-43,54)	16,33 (26,99-35,38)	**0.002**
HG/SMM dominant	0,99 (1,22-1,39)	0,57 (0,82-1,1)	**0.000**
HG/SMM recessive	0,88 (1,06-1,36)	0,48 (0,81-1,11)	**0.001**
HG/ASMM dominant	1,29 (1,6-1,77)	0,74 (1,06-1,4)	**0.000**
HG/ASMM recessive	1,15 (1,38-1,66)	0,615 (1,03-1,41)	**0.001**
HG/BMI dominant	1,09 (1,48-1,79)	0,71 (1,08-1,36)	**0.000**
HG/BMI recessive	1,01(1,25-1,65)	0,66 (1,05-1,32)	**0.004**

C.

	Intolerants	Tolerants	P
Age	60 (65-71)	58 (62-66,5)	
HG dominant (Kg)	13,61 (18,14-20,87)	18,14 (30,39 41,73)	**0.000**
HG recessive (Kg)	11,79 (14,51-17,24)	17,24 (27,22-39,46)	**0.000**
HG/SMM dominant	0,80 (0,91-1,05)	1,05 (1,53-2,17)	**0.000**
HG/SMM recessive	0,62 (0,73-0,89)	0,91 (1,38-1,98)	**0.000**
HG/ASMM dominant	0,91 (1,02-1,22)	1,25 (1,87-2,64)	**0.000**
HG/ASMM recessive	0,70 (0,87-1,01)	1,13 (1,68-2,2)	**0.000**
HG/BMI dominant	0,52 (0,67-0,76)	0,81 (1,35-1,83)	**0.000**
HG/BMI recessive	0,42 (0,55-0,65)	0,72 (1,06-1,65)	**0.000**

Supplementary material Table S3. Differences in HG and MQi between dominant and non-dominant arms. Results are expressed as median and IQR values. P= Kruskal-Wallis test. A: results in the whole population. B: results in Men; C: results in Women; Legend: HG: Hand Grip; SMM: Skeletal Muscle Mass; ASMM: appendicular skeletal muscle mass; BMI: body mass index
